# Prognostic Significance of B7H3 Expression in Solid Tumors: A Systematic Review and Meta-Analysis

**DOI:** 10.3390/ijms26073044

**Published:** 2025-03-26

**Authors:** Sylwia Mielcarska, Agnieszka Kula, Miriam Dawidowicz, Dariusz Waniczek, Elżbieta Świętochowska

**Affiliations:** 1 Department of Medical and Molecular Biology, Faculty of Medical Sciences in Zabrze, Medical University of Silesia in Katowice, 19 Jordana St., 41-800 Zabrze, Poland; 2 Department of Oncological Surgery, Faculty of Medical Sciences in Zabrze, Medical University of Silesia, 41-514 Katowice, Poland; d201070@365.sum.edu.pl (A.K.); d201069@365.sum.edu.pl (M.D.); dwaniczek@sum.edu.pl (D.W.)

**Keywords:** B7H3, solid tumors, meta-analysis, overall survival (OS), progression-free survival (PFS), recurrence-free survival (RFS), disease-free survival (DFS), disease-specific survival (DSS)

## Abstract

B7H3 (CD276), an immunoregulatory molecule known for its role in immune evasion by transmitting inhibitory signals to T lymphocytes, has garnered significant attention in recent years as a promising target for cancer immunotherapy. This interest is largely due to its high expression in various types of solid tumors, coupled with low protein levels in normal tissues. However, studies examining the impact of B7H3 on survival outcomes have shown inconsistent results, leaving its prognostic significance not fully clarified. Therefore, this meta-analysis aimed to assess the relationship between B7H3 expression and various prognostic parameters in patients with solid malignancies. PubMed, Web of Science (WOS), Cochrane, SCOPUS, and Embase databases were searched for eligible articles published until November 2024. Statistical analysis was performed using R studio (version 4.3.2). The analysis included a total of 51 eligible studies comprising 11,135 patients. Results showed that overexpression of B7H3 is a negative predictor for all examined survival outcomes: OS (HR = 1.71, 95% CI = 1.44–2.03, *p* < 0.0001), DFS (HR = 2.02, 95% CI = 1.49–2.73, *p* < 0.0001), PFS (HR = 2.10, 95% CI = 1.44–3.06, *p* < 0.0001), RFS (HR = 1.66, 95% CI = 1.11–2.48, *p* = 0.01), and DSS (HR = 1.70, 95% CI = 1.24–2.32, *p* < 0.01). Despite the high heterogeneity observed across the studies, the sensitivity analysis confirmed the robustness of these results. This research suggests that B7H3 may serve as an effective biomarker for prognosis in solid tumors.

## 1. Introduction

Immune Checkpoint Inhibitors (ICBs) are designed to block immune checkpoint molecules from binding to co-receptors, thereby disrupting immune suppression and promoting an anti-tumor response by inhibiting the signals that suppress T cells [1]. This targeted approach has significantly changed the face of treatment for tumor types, leading to notable improvements in patient survival [2].

Nevertheless, there are limitations to the effectiveness of the most commonly used ICBs, particularly those that target the PD-1/PD-L1 axis. Consequently, there is an ongoing need to explore new potential targets for immunotherapy [3]. Current research is focused on identifying novel molecules and pathways to enhance treatment responses and broaden the patient population that may benefit from targeted therapies.

B7-Homolog 3 (B7H3) is an immune checkpoint protein from the B7 family, first described in 2001. It functions as an immunoregulatory protein with both costimulatory and coinhibitory properties, playing a dual role in immunity [4]. B7H3 is highly expressed in cancer and immune cells, and it is also involved in non-immune processes, including cancer cell proliferation, metastasis, and treatment resistance [5]. The upregulation of B7H3 expression has been observed in various malignant neoplasms, including non-small cell lung cancer (NSCLC), renal, urothelial, prostate, gastric, pancreatic, colorectal, and ovarian cancers, as well as osteosarcoma, head and neck squamous carcinoma, and glioblastoma. B7H3 has garnered significant attention due to its high selective expression across a range of tumors, accompanied by a very low expression level in normal tissues. This characteristic suggests that its targeting with therapeutic agents could lead to cancer-specific toxicity while minimizing damage to non-cancerous cells [5,6]. In addition to tumor cells, B7H3 was found in immune cells, including dendritic cells, myeloid-derived suppressor cells (MDSCs), neutrophils, monocytes, macrophages, B cells, and activated T lymphocytes. Low levels of B7H3 protein expression have been observed in a limited number of non-cancerous tissues, including epithelial cells, pleural effusions, and serum [4,5]. The primary challenge in targeting B7H3 with pharmacological agents is the absence of a clearly identified B7H3 receptor. CD4+ and CD8+ T cells are suspected to express the B7H3 receptor, which binds to B7H3 expressed by cancer cells and Antigen Presenting Cells (APCs) [6]. Clinical trials investigating inhibitors targeting B7H3 report promising preliminary results across various malignancies; however, most of them are still ongoing [7].

Despite numerous studies demonstrating an association between the upregulation of B7H3 expression and poor prognosis and higher recurrence rates, the prognostic potential of B7H3 needs further clarification regarding different cancer types, patient populations, and methods for measuring B7H3 expression. We performed a meta-analysis to assess the relationship between the upregulation of B7H3 expression and survival outcomes in patients with solid cancers. This research evaluates the prognostic significance of B7H3 on overall survival (OS), disease-specific survival (DSS), disease-free survival (DFS), progression-free survival (PFS), and recurrence-free survival (RFS).

## 2. Materials and Methods

### 2.1. Meta-Analysis

#### 2.1.1. Search Strategy for Meta-Analysis and Clinical Outcomes

The systematic review and meta-analysis were conducted following the Preferred Reporting Items for Systematic Reviews and Meta-Analyses (PRISMA) guidelines. The protocol for this review was published and registered with the PROSPERO platform (registration ID CRD42024590645). We systematically searched the following databases for relevant studies examining the prognostic potential of B7H3 expression in solid cancers: PubMed (accessed on 1 October 2024), Embase (accessed on 1 October 2024), Cochrane (accessed on 1 October 2024), Web of Science (accessed on 1 October 2024), and Scopus (accessed on 1 October 2024). Only articles published before 15 November 2024, were included in the research. Articles were identified using search terms that included “B7H3” OR “B7H3” OR “CD276” AND “cancer” AND “carcinoma” AND “malignancy” AND “tumor” AND “neoplasms”. Cancer survival parameters included overall survival (OS), disease-free survival (DFS), recurrence-free survival (RFS), progression-free survival (PFS), and disease-specific survival (DSS).

#### 2.1.2. Inclusion and Exclusion Criteria

The criteria for inclusion were outlined as follows: Patients diagnosed with solid tumors prior to enrollment were eligible for the study. Only randomized controlled trials (RCTs) and observational studies were included. B7H3 expression needed to be assessed using immunohistochemistry (IHC), and a survival outcome had to be reported either as a hazard ratio (HR) or through a Kaplan–Meier curve, which must include a number-at-risk table if the HR was not available.

The exclusion criteria encompassed studies focused on non-solid tumors, those assessing B7H3 expression by methods other than IHC, and studies that reported survival outcomes exclusively through Kaplan–Meier curves without a corresponding number-at-risk table. Additionally, case reports, single-cell sequencing data, animal experiments, articles based on The Cancer Genome Atlas (TCGA) or other publicly available platforms, meta-analyses, network meta-analyses, reviews, conference papers, and study protocols were also excluded.

#### 2.1.3. Study Selection and Data Extraction

The review was conducted by two independent reviewers, SM and AK, who assessed the titles and abstracts of studies that met the eligibility criteria. In the subsequent stage, the full texts of the studies with sufficient data for inclusion were downloaded. The following data were extracted by the two authors (S.M. and A.K.): the title of the study, first authors, year of publication, sample size, cancer type, information about B7H3 expression (including localization and the cutoff value for distinguishing high B7H3 expression), and clinical outcomes (overall survival, progression-free survival, recurrence-free survival, disease-free survival, and disease-specific survival). Additionally, details about the evaluation of B7H3 by immunohistochemistry (IHC) were included, along with a quality assessment and methods used for hazard ratio (HR) estimation (either univariate or multivariate). If the HR was not available, it was calculated indirectly from Kaplan–Meier curves and the number-at-risk table using WebPlotDigitizer v4.7, employing the method created by Guyot P. in R Studio [8]. Trials that presented only Kaplan–Meier curves without accompanying number-at-risk tables were excluded from the review. In cases of discrepancies between the main reviewers, a third author (M.D.) was consulted.

#### 2.1.4. Strategy for Meta-Analysis

The meta-analysis was conducted using R software (version 4.0.3). A multivariate model was employed to estimate the hazard ratio (HR); if a multivariate model was unavailable, a univariate model was utilized instead. To evaluate statistical heterogeneity, both the chi-square test and the I^2^ statistic were applied. According to J.P. Higgins and Thompson (2002), an I^2^ value above 75% indicates high heterogeneity, 50–75% indicates moderate heterogeneity, and 25–50% indicates low heterogeneity [9]. An I^2^ value below 50% and a *p*-value greater than 0.05 suggest no substantial heterogeneity among studies, allowing for the use of a fixed-effect model to pool HR with a 95% confidence interval. Conversely, if the I^2^ value exceeded 50% and the *p*-value was below 0.05, a random-effect model was employed due to significant heterogeneity.

Subgroup and sensitivity analyses were conducted to explore the sources of heterogeneity. The sensitivity analysis involved excluding one study at a time to check the influence of a particular study on the outcome and I^2^. Due to high heterogeneity in the meta-analysis pooling HR for OS in solid tumors and gastrointestinal malignancies, which was not explained by subgroup analysis, we employed Graphic Display of Heterogeneity (GOSH) plot analysis available in the dmetar (R package version 0.1.0). In GOSH analysis, the same meta-analysis model is fitted to all possible subsets of included studies, providing a model for 2^K−1^ possible combinations, while sensitivity analysis allows fitting only K models [10]. The obtained models are plotted with the pooling HR on the x-axis and the between-study heterogeneity on the y-axis to find specific patterns of heterogeneity. Next, we used the K-means algorithm in the GOSH diagnostics function to identify heterogeneity patterns and detect studies that mostly influence the cluster make-up [10]. Finally, we performed OS meta-analyses for all solid tumors and gastrointestinal cancers by removing studies identified by the K-mean algorithm. Funnel plots and Begg’s and Egger’s tests were used to evaluate publication bias. *p*-values ≤ 0.05 were considered significant.

#### 2.1.5. Quality Assessment

The quality of the included studies was independently evaluated by two reviewers (AK and MD) using the Newcastle–Ottawa Quality Assessment Scale (NOS). This scale assesses the quality of the included trials based on three criteria: selection, comparability, and exposure. The total score can range from 0 to 9 points.

Although overall survival (OS) is the most commonly reported measure of survival outcomes, it is not considered an indicator of study quality due to a high risk of bias. Studies that presented only OS received one star, while those that reported additional survival parameters (such as DFS, RFS, and DSS) received two stars [11].

In the comparability section, one star was awarded to studies that presented hazard ratios (HR) obtained through univariate analysis, and two stars were given to studies reporting HR from a multivariate model. Studies that received a score above six points were classified as high quality.

## 3. Results

### 3.1. Search Results

**Figure 1:**
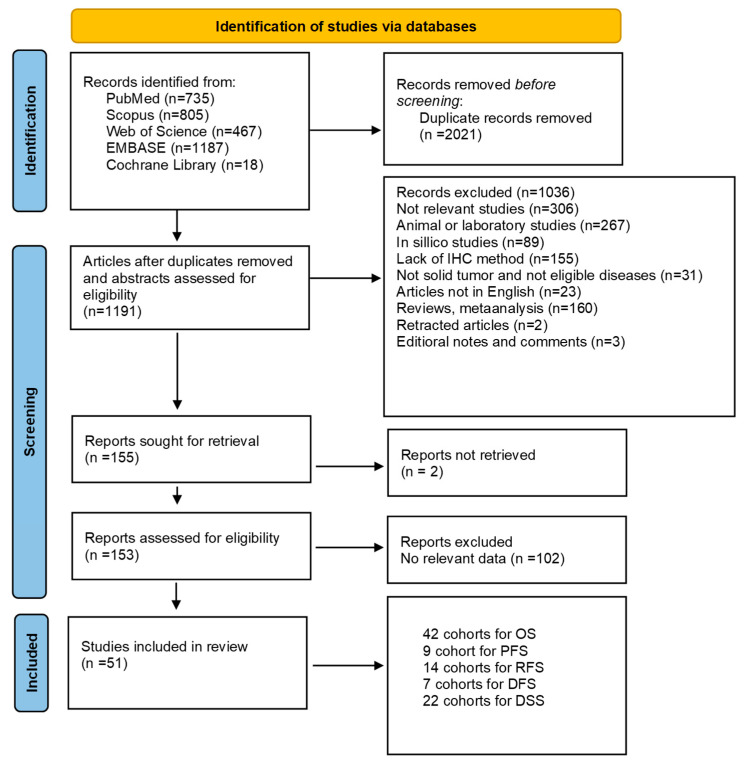
PRISMA 2020 flow diagram of study selection and inclusion.

Figure 1 presents a flowchart that summarizes the selection procedure and literature search strategy. Initially, 3212 records were found through database research. In the following step, 2021 records were excluded due to duplicates, leaving 1191 articles to be evaluated by reviewing their titles and abstracts. At this stage, 1036 articles were excluded. Subsequently, 155 studies were assessed for eligibility, and ultimately, 51 articles met the inclusion criteria and were included in the meta-analysis.

### 3.2. Study Characteristics

The main characteristics of the included studies are summarized in Table 1. A total of 51 articles were published between 2007 and 2024, involving 11,135 individuals with solid tumors. All studies were retrospective cohort studies that reported both univariate and multivariate models. When both types of hazard ratios (HRs) were available, the multivariate HR was used to minimize bias. The majority of studies provided an HR; only in two studies were HRs recalculated from Kaplan–Meier curves and number-at-risk tables [12,13].

In all studies, immunohistochemistry was employed to measure B7H3 expression; however, the methods of evaluation and the cut-off values for categorizing high/positive versus low/negative expression varied significantly among the studies. The populations studied were drawn from various countries, including China, Japan, South Korea, Australia, the United States, the United Kingdom, Norway, Iran, and the Netherlands. Patients were diagnosed with the following types of tumors: PCa—prostate cancer, OC—ovarian cancer, CRC—colorectal cancer, BC—breast cancer, NSCLC—non-small-cell lung carcinoma, GC—gastric cancer, LUAD—lung adenocarcinoma, EC—endometrial cancer, ATC—anaplastic thyroid cancer, PDTC—poorly differentiated thyroid carcinoma, PDAC—pancreatic ductal adenocarcinoma, UTUC—upper tract urothelial carcinoma, ACC—adrenocortical carcinoma, ICC—intrahepatic cholangiocarcinoma, HCC—hepatocellular carcinoma, ccRCC—clear cell renal cell carcinoma, UCC—urothelial cell carcinoma, HNSCC—head and neck squamous cell carcinoma, NB—neuroblastoma, PTC—papillary thyroid carcinoma, BLCA—bladder urothelial carcinoma, GBC—gallbladder cancer, CC—cervical cancer, GEA—gastroesophageal adenocarcinoma, SC—spinal chondroma, OS—osteosarcoma, CP—craniopharyngioma, and AAC—ampullary adenocarcinoma. Data from 12 studies was extracted to perform analysis for gastrointestinal tumors. Among survival outcomes, OS was reported by 42 studies, PFS by 9, RFS by 14, DFS by 7, and DSS by 22. The NOS scores for all qualified studies were above 6. Full scoring on the NOS is shown in Table 1.

### 3.3. Meta-Analysis Results

#### 3.3.1. B7H3 Expression and Overall Survival

**Figure 2:**
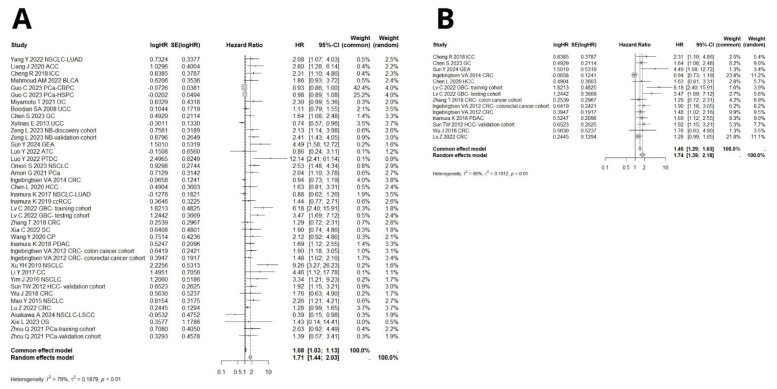
(**A**) Forest plots of studies exploring hazard ratios of B7H3 for overall survival in solid cancers, pooled by random effect model due to high heterogeneity among studies (I^2^ = 79%, 95% CI [71.6–84.2%]). In total, data from 6928 patients were analyzed [12,13,14,15,16,19,20,21,23,24,25,29,30,33,34,35,38,40,41,42,44,45,46,47,48,49,50,51,52,55,56,57,58,61]. (**B**) Forest plots of studies exploring hazard ratios of B7H3 for overall survival in gastrointestinal cancers, pooled by random effect model due to substantial heterogeneity among studies (I^2^ = 66%, 95% CI [39.4–80.5%]). In total, data from 3212 patients were analyzed [13,16,19,30,33,45,46,49,50,56,58,61].

Data from 40 cohorts were analyzed to explore the association between B7H3 expression and overall survival. The random pooled hazard ratio indicated that high B7H3 expression was associated with shorter OS in both all solid tumors (HR = 1.71, 95% CI [1.44–2.03], *p* < 0.0001, Figure 2A) and gastrointestinal cancers (HR = 1.74, 95% CI [1.39–2.18], *p* < 0.0001, Figure 2B). Additionally, significant heterogeneity was observed among the studies, with I^2^ values of 79% (95% CI [71.6–84.2%], *p* < 0.0001) for all solid tumors and 66% (95% CI [39.4–80.5%], *p* < 0.001) for gastrointestinal cancers. Therefore, a random effects model was used to estimate the pooled HR.

**Figure 3:**
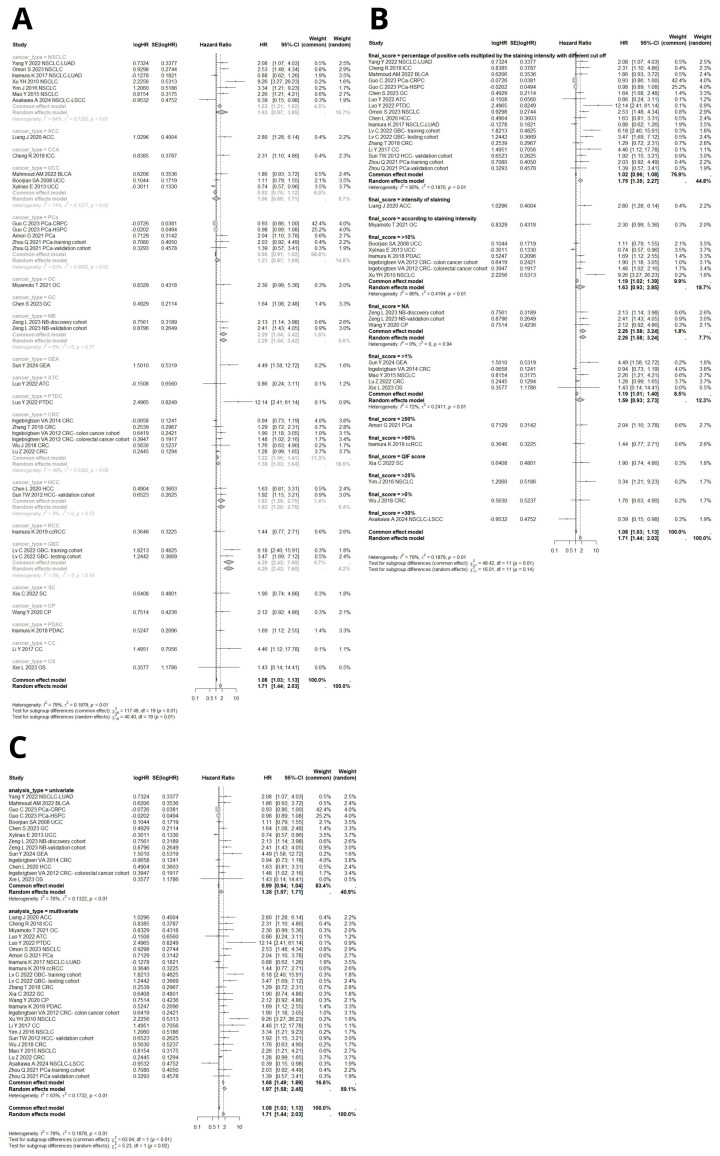
(**A**) Forest plot of the subgroup analysis for B7H3 and OS, stratified by cancer type [12,13,14,15,16,19,20,21,23,24,25,29,30,33,34,35,38,40,41,42,44,45,46,47,48,49,50,51,52,55,56,57,58,61]. (**B**) Forest plot of the subgroup analysis for B7H3 and OS, stratified by cutoff value for high and low B7H3 expression [12,13,14,15,16,19,20,21,23,24,25,29,30,33,34,35,38,40,41,42,44,45,46,47,48,49,50,51,52,55,56,57,58,61]. (**C**) Forest plot of the subgroup analysis for B7H3 and OS, stratified by different models to estimate HR [12,13,14,15,16,19,20,21,23,24,25,29,30,33,34,35,38,40,41,42,44,45,46,47,48,49,50,51,52,55,56,57,58,61].

Subgroup analyses were conducted to identify potential causes of heterogeneity. Stratifying by tumor type revealed high heterogeneity both between and within groups (*p* < 0.0001, Figure 3A). The highest heterogeneity was found in the non-small cell lung cancer (NSCLC) group, which included the largest number of studies (k = 7, I^2^ = 84%, *p* < 0.01, Figure 3A). Among cancer types represented by at least two cohorts, high B7H3 expression was linked to poor OS in neuroblastoma (HR = 2.29, 95% CI [1.54–3.42]), colorectal cancer (HR = 1.30, 95% CI [1.03–1.64]), hepatocellular carcinoma (HR = 1.82, 95% CI [1.20–2.75]), and gallbladder cancer (HR = 4.29, 95% CI [2.42–7.60]). No significant association between B7H3 expression and OS was found in NSCLC, UCC, and PCa (Figure 3A).

**Figure 4:**
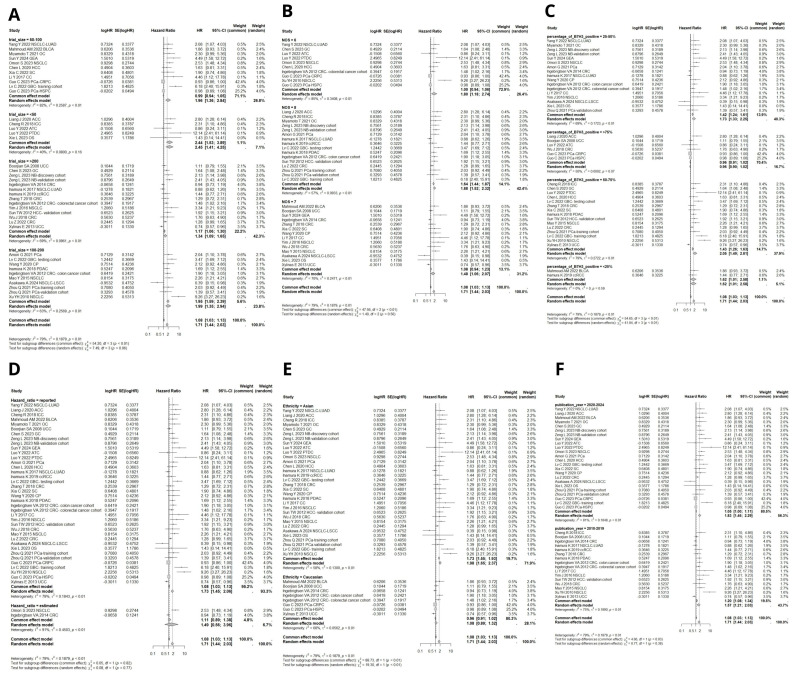
(**A**) Forest plot of the subgroup analysis for B7H3 and OS, stratified by sample size [12,13,14,15,16,19,20,21,23,24,25,29,30,33,34,35,38,40,41,42,44,45,46,47,48,49,50,51,52,55,56,57,58,61]. (**B**) Forest plot of the subgroup analysis for B7H3 and OS, stratified by studies quality in NOS [12,13,14,15,16,19,20,21,23,24,25,29,30,33,34,35,38,40,41,42,44,45,46,47,48,49,50,51,52,55,56,57,58,61]. (**C**) Forest plot of the subgroup analysis for B7H3 and OS, stratified by the percentage of B7H3 positive cases in each study [12,13,14,15,16,19,20,21,23,24,25,29,30,33,34,35,38,40,41,42,44,45,46,47,48,49,50,51,52,55,56,57,58,61]. (**D**) Forest plot of the subgroup analysis for B7H3 and OS, stratified by method for obtaining HR (reported vs. recalculated) [12,13,14,15,16,19,20,21,23,24,25,29,30,33,34,35,38,40,41,42,44,45,46,47,48,49,50,51,52,55,56,57,58,61]. (**E**) Forest plot of the subgroup analysis for B7H3 and OS, stratified by ethnicity (Asian vs. Caucasian) [12,13,14,15,16,19,20,21,23,24,25,29,30,33,34,35,38,40,41,42,44,45,46,47,48,49,50,51,52,55,56,57,58,61]. (**F**) Forest plot of the subgroup analysis for B7H3 and OS, stratified by publication year [12,13,14,15,16,19,20,21,23,24,25,29,30,33,34,35,38,40,41,42,44,45,46,47,48,49,50,51,52,55,56,57,58,61].

Further subgroup analyses were performed based on the cut-off values for high and low B7H3 expression (Figure 3B), the methods used to estimate hazard ratios (HR) (Figure 3C), and the sample sizes (Figure 4A). The analyses also considered population ethnicity, the quality of studies assessed using the Newcastle–Ottawa Scale (NOS), the publication year (before and after 2020), and the methods for obtaining HR (whether reported or recalculated from the Kaplan–Meier curve) (Figure 4B–F). The B7H3 thresholds varied significantly across studies due to the lack of standardized methods for immunohistochemistry evaluation. In the subgroup analysis by cut-off value, significant intra- and inter-study heterogeneity was observed (*p* < 0.0001). Studies that assessed B7H3 expression with cut-offs of >1% or >10% of stained tumor cells did not support a significant association between B7H3 and reduced OS (HR = 1.59, 95% CI [0.93–2.73] and HR = 1.63, 95% CI [0.93–2.85], respectively, Figure 3B). Importantly, the majority of studies (n = 18) estimated B7H3 expression as a percentage of positive cells multiplied by the intensity of staining with various cut-off values; this method exhibited high heterogeneity (I^2^ = 80%) and was associated with poorer OS (HR = 1.75, 95% CI [1.35–2.27], Figure 3B). For two studies, cut-off values were not available.

The varying cut-off values for categorizing high and low B7H3 expression across the included studies rendered subgroup analyses inconclusive. To address this, we conducted additional subgroup analyses stratified by the percentage of cases positive for B7H3 expression in each study, which closely relates to the established cut-off values. Notably, in the subgroup with a B7H3 positivity rate exceeding 75%, the expression of B7H3 did not show a significant association with OS (Figure 4C). The highest heterogeneity was observed in the subgroup with a B7H3 positivity rate between 50% and 75% (I^2^ = 79%); however, high heterogeneity persisted in other groups as well, including the subgroup with less than 25% positivity, which comprised only two studies.

Subgroup comparisons based on ethnic populations indicated that the upregulation of B7H3 expression was linked to reduced OS exclusively in Asian populations, while no such association was observed in Caucasian populations. (HR = 1.98, 95% CI [1.65–2.37] vs. HR = 1.08, 95% CI [0.89–1.32], Figure 4E). Considering the method used for HR estimation, multivariate models were more commonly reported and had a greater impact on overall outcomes compared to univariate analyses (HR = 1.71, 95% CI [1.44–2.03] vs. HR = 1.35, 95% CI [1.07–1.71], *p* = 0.02, Figure 3C). Both studies directly reported hazard ratios (HR). Those in which HR was derived from Kaplan–Meier curves indicated a positive correlation between elevated B7H3 levels and shorter overall survival (OS) (Figure 4D). Subgroup analysis based on sample size revealed that studies with the smallest sample sizes (<50 cases) had the highest pooled HR (HR = 2.45, 95% CI [1.41–4.25]). A significant impact of B7H3 on reduced survival was observed across all groups (Figure 4A).

Evaluating study quality using the Newcastle–Ottawa Scale (NOS), we found that studies with lower NOS scores exhibited higher heterogeneity (NOS 6, HR = 1.80, 95% CI [1.18–2.74], I^2^ = 85% vs. NOS 8, HR = 1.88, 95% CI [1.52–2.32], I^2^ = 57%, Figure 4B).

We performed a subgroup analysis based on publication year, categorizing research into studies published before and after 2020. In both groups, B7H3 was associated with poorer OS outcomes (2020–2024: HR = 1.83, 95% CI [1.46–2.29], I^2^ = 81% vs. 2010–2019: HR = 1.57, 95% CI [1.21–2.03], I^2^ = 75%, Figure 4F). The primary characteristics of the included studies are presented in Table 1. A total of 51 articles were published between 2007 and 2024, involving 11,135 individuals with solid tumors. All studies were retrospective cohort studies that reported both univariate and multivariate models. When both types of hazard ratios (HRs) were available, the multivariate HR was used to minimize bias.

#### 3.3.2. B7H3 Expression and Its Relationship with DFS, PFS, RFS, and DSS

**Figure 5:**
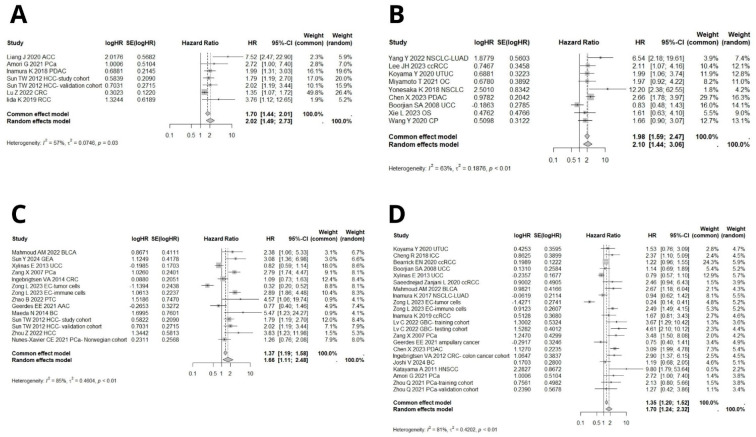
(**A**) Forest plot of studies exploring hazard ratios of B7H3 for disease-free survival in solid tumors, pooled by random effect model due to moderate heterogeneity among studies (I^2^ = 66%, 95% CI [0.0–81.3%]). Data from a total of 1670 patients was analyzed [20,29,33,56,58,60]. (**B**) Forest plots of studies exploring hazard ratios of B7H3 for progression-free survival in solid tumors, pooled by random effect model due to moderate heterogeneity among studies (I^2^ = 63%, 95% CI [24,1–82.0%]). A total of data from 1436 patients was analyzed [15,18,25,26,27,28,34,55,57]. (**C**) Forest plot of studies exploring hazard ratios of B7H3 for recurrence-free survival in solid tumors, pooled by random effect model due to high heterogeneity (I^2^ = 85%, 95% CI [76.6–90.6%]). A total of data from 4360 patients was analyzed [13,22,31,33,35,39,43,44,50,53,54,59]. (**D**) Forest plots of studies exploring hazard ratios of B7H3 for disease-specific survival in solid tumors, pooled by random effect model due to high heterogeneity (I^2^ = 81%, 95% CI [72.8–87.3%]). A total of data from 5764 patients was analyzed [17,20,27,28,30,32,34,35,36,37,42,44,45,46,54,58,59].

Seven cohorts provided suitable data for the DFS analysis. Due to significant heterogeneity, a random effects model was employed to pool the effect size. High B7H3 expression was associated with decreased DFS (HR = 2.02, 95% CI [1.49–2.73], *p* < 0.0001, Figure 5A). Nine studies investigated the relationship between B7H3 expression and PFS. Due to significant heterogeneity, the data were analyzed using a random-effects model. Upregulation of B7H3 expression indicated poor PFS (HR = 2.10, 95% CI [1.44–3.06], *p* < 0.0001, Figure 5A).

Fourteen included cohorts provided sufficient data for RFS analysis. Due to high heterogeneity, a random effects model was used to calculate pooled HR. High B7H3 expression was linked with worser RFS (HR = 1.66, 95% CI [1.11–2.48], *p* = 0.01, Figure 5C).

Twenty-two studies analyzed disease-specific survival (DSS) in relation to B7H3 expression. Due to high heterogeneity, a random effects model was applied to pool hazard ratios (HR). The combined results demonstrated a negative association between B7H3 upregulation and DSS (Figure 5D).

### 3.4. Publication Bias and Sensitivity Analysis

**Figure 6:**
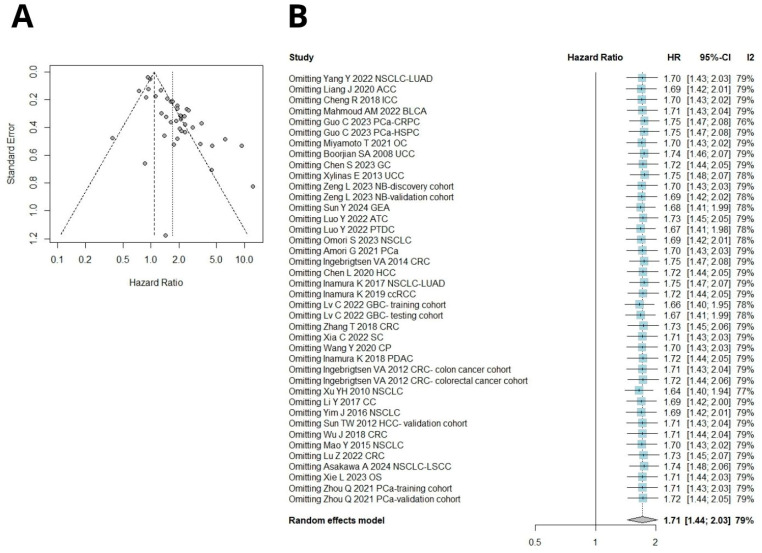
(**A**) Begg’s funnel plot of publication biases on all evaluated studies between B7H3 and OS. Egger test *p* < 0.0001, Begg’s test *p* = 0.07. (**B**) One leave meta-analysis for assessing the influence of excluding a particular study on pooled HR for OS [12,13,14,15,16,19,20,21,23,24,25,29,30,33,34,35,38,40,41,42,44,45,46,47,48,49,50,51,52,55,56,57,58,61].

Begg’s funnel plot and Egger’s test were used to assess publication bias. Included cohorts evaluating OS yielded significant Egger’s test results (*p* = 0.07, *p* < 0.0001 for Begg’s and Egger’s tests, respectively, Figure 6A). Sensitivity analysis showed that excluding any particular study did not significantly influence the pooled HR, and high B7H3 expression still predicted poorer OS (Figure 6B).

**Figure 7:**
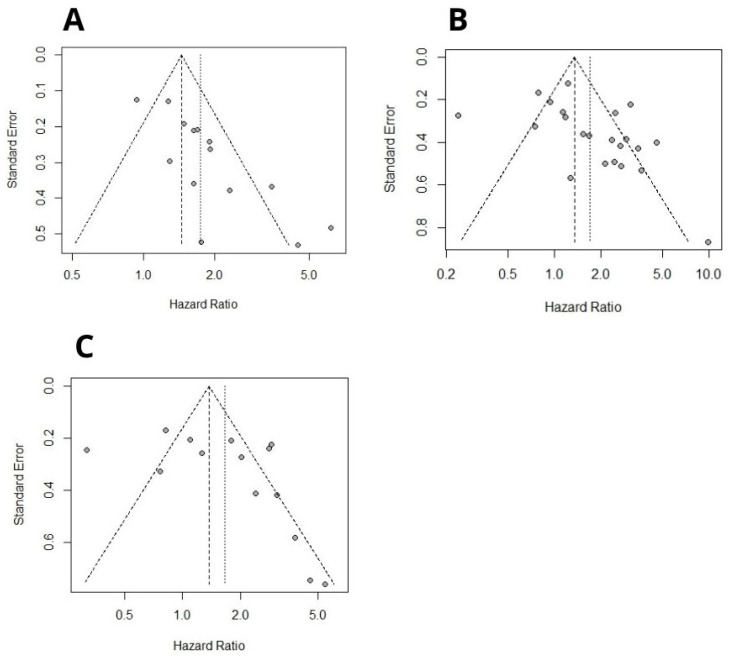
(**A**) Begg’s funnel plot of publication biases on all evaluated studies between B7H3 and OS in gastrointestinal cancers. Egger test *p* = 0.0003, Begg’s test *p* = 0.007. (**B**) Begg’s funnel plot of publication biases on all evaluated studies between B7H3 and DSS in solid tumors. Egger test *p* = 0.034, Begg’s test *p* = 0.034). (**C**) Begg’s funnel plot of publication biases on all evaluated studies between B7H3 and RFS in solid tumors. Egger test *p* = 0.14, Begg’s test *p* = 0.17).

**Figure 8:**
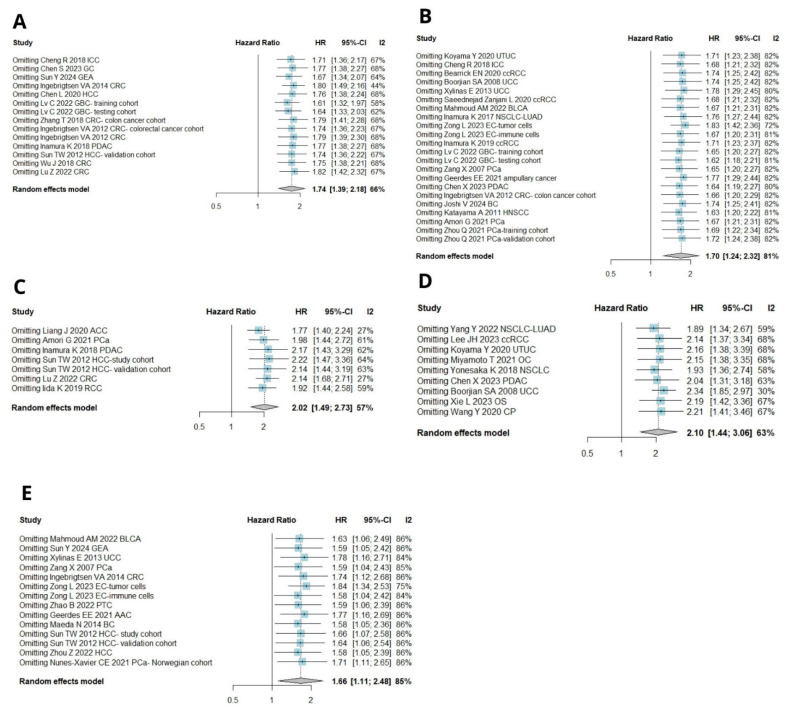
(**A**) One-leave meta-analysis for assessing the influence of excluding a particular study on pooled HR for OS in gastrointestinal cancers [13,16,19,30,33,45,46,49,50,56,58,61]. (**B**) One-leave meta-analysis for assessing the influence of excluding a particular study on pooled HR for DSS in solid tumors [17,20,27,28,30,32,34,35,36,37,42,44,45,46,54,58,59]. (**C**) One-leave meta-analysis to assess the influence of excluding a particular study on pooled HR for DFS in solid tumors [20,29,33,56,58,60]. (**D**) One-leave meta-analysis for assessing the influence of excluding a particular study on pooled HR for PFS in solid tumors [15,18,25,26,27,28,34,55,57]. (**E**) One-leave meta-analysis for assessing the influence of excluding a particular study on pooled HR for RFS in solid tumors [13,22,31,33,35,39,43,44,50,53,54,59].

Similarly, studies investigating OS in gastrointestinal cancers and evaluating DSS yielded significant Begg’s and Egger’s test results (Figure 7A,B). Sensitivity analysis also confirmed the stability of the results, as the removal of any single study did not alter the significance of pooled HRs (Figure 8A,B). No publication bias was detected for RFS (Figure 7C). Due to a number of included studies <10, tests for funnel plot asymmetry were not conducted for DFS and PFS. One leave meta-analysis performed for DFS, PFS, and RFS showed that any individual cohort did not affect the association between B7H3 and survival outcomes (Figure 8C–E).

### 3.5. GOSH Analysis

**Figure 9:**
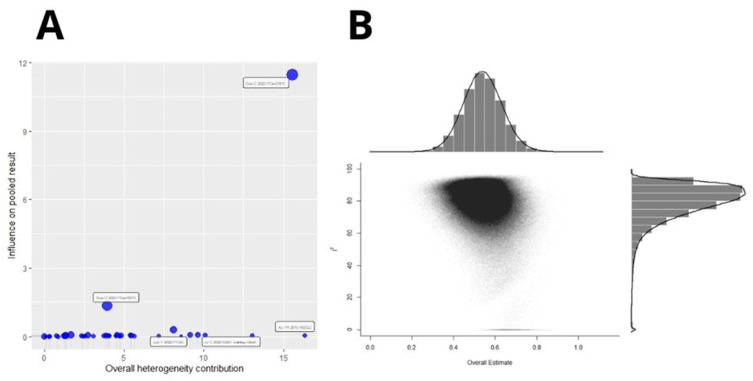
(**A**) Diagnostics of between-study heterogeneity on a Baujat plot. (**B**) GOSH plot analysis. To elucidate patterns of heterogeneity, the same meta-analysis model was fitted to all possible subsets of included studies.

**Figure 10:**
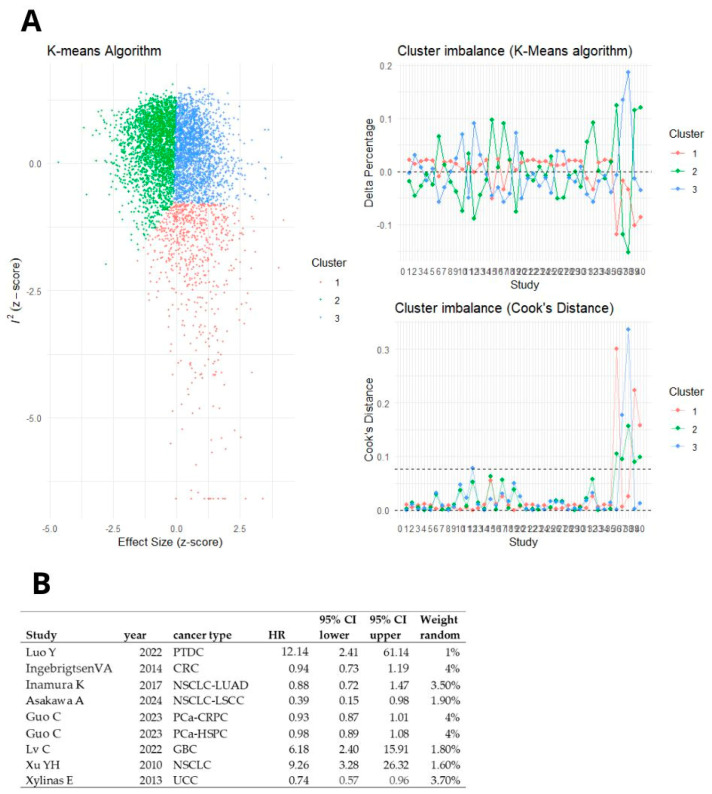
(**A**) Outliers identified using the K-means algorithm. (**B**) Studies and patients cohorts identified as outliners by K-means algorithm [13,14,21,23,24,35,41,46].

**Figure 11:**
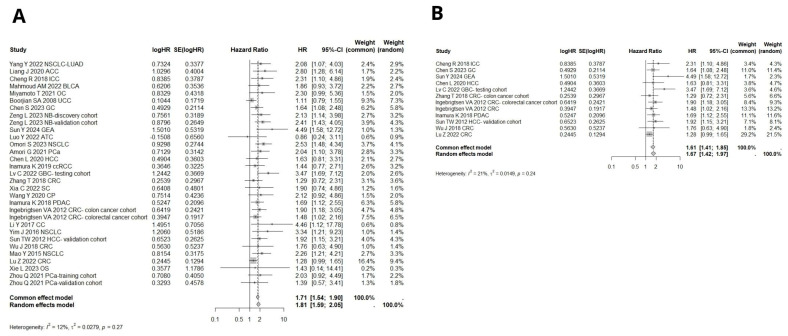
(**A**) Forest plot of studies exploring hazard ratios of B7H3 for overall survival in solid cancers, after excluding potential outliers through the K-means algorithm in a GOSH analysis [12,15,16,19,20,25,29,30,33,34,38,40,42,44,45,47,48,49,50,51,52,55,56,57,58,61]. (**B**) Forest plot of studies exploring hazard ratios of B7H3 for overall survival in gastrointestinal cancers, after excluding potential outliers through the K-means algorithm in a GOSH analysis [16,19,30,33,45,46,49,50,56,58,61].

As subgroup and sensitivity analyses did not clarify the source of heterogeneity, we conducted Baujat and GOSH plot analyses to identify studies influencing the heterogeneity (Figure 9). The GOSH plot analysis, utilizing the K-means algorithm, revealed that the studies by Luo Y., Ingebrigtsen VA, Inamura K. (2017), Asakawa A., Guo C., Lv C., Xu YH, and Xylinas E. were potential outliers (Figure 10). A meta-analyses for overall survival (OS) in solid cancers and gastrointestinal tumors were performed after excluding these outlier studies (Figure 11). The pooled hazard ratios were similar; however, the heterogeneity decreased to 12% for solid cancers and to 21% for gastrointestinal cancers (Figure 11).

## 4. Discussion

Immune checkpoints play a pivotal role in maintaining the balance between self-tolerance to autoantigens and the efficient recognition and elimination of allogenic proteins facilitated by adaptive immunity [62]. The use of immunotherapies has revolutionized the treatment of various solid tumors. However, their effectiveness may be limited for certain types of cancer, and they can lead to significant organ-specific toxicity [63]. Additionally, increasing drug resistance is another challenge in the immunotherapy field [63]. Therefore, exploring new immune checkpoints is essential to increase the number of patients who can benefit from immunotherapy. Like other members of the B7 family, B7H3 is expressed by cancer cells to evade T cells and NK cells immune surveillance. It also modifies cytokine secretion, promotes the polarization of macrophages into the M2 phenotype, and enhances the proliferation and migration of cancer-associated fibroblasts (CAFs). These actions contribute to an immunosuppressive tumor microenvironment. Additionally, B7H3 has been found to support tumor progression through non-immunological processes, including the proliferation, invasiveness, and migration of cancer cells, epithelial-to-mesenchymal transition (EMT), remodeling of the extracellular matrix, and tumor angiogenesis [6]. Taken together, the biological functions of B7H3 are associated with higher tumor aggressiveness and, consequently, poor prognosis for patients. One of the primary challenges in fully understanding the role of B7H3 in tumor progression, as well as in predicting the most significant toxicities associated with B7H3 inhibition, is the absence of fully identified B7H3 receptors. Several candidate molecules have been proposed as potential binding partners for B7H3, including triggering receptor expressed on myeloid cells (TREM)-like transcript 2 (TLT-2, TREML2), interleukin-20 receptor subunit alpha (IL20RA), phospholipase A2 receptor 1 (PLA2R1), 4-1BB, and AAMP [64,65,66]. These molecules have demonstrated both costimulatory and coinhibitory activity in immune responses, and their interactions with B7H3 continue to be the subject of extensive investigation. The unclear nature of B7H3 receptors, which may exert opposite effects depending on the immune cells expressing them, could lead to conflicting findings regarding B7H3′s role in certain malignancies. Among the studies conducted, only one, conducted by Asakawa in a lung squamous cell carcinoma (LSCC) cohort, reported that patients with high B7H3 expression experienced prolonged survival [41]. This finding suggests a positive prognostic potential and possible antitumoral activity of B7H3 in this particular cancer type. Most included studies indicated a positive correlation between B7H3 upregulation and reduced survival or showed no significant relationship between B7H3 and patient outcomes. B7H3 has been shown to be highly upregulated in the majority of the most common solid tumors while exhibiting very low protein expression in non-cancerous tissues and immune cells. This suggests that targeting B7H3 could lead to fewer immune-related adverse effects. For this reason, inhibiting B7H3 through the use of CAR T cells, antibody-dependent cellular cytotoxicity (ADCC), and monoclonal antibodies is being extensively evaluated in clinical trials. A phase I clinical trial of the B7H3 blocking monoclonal antibody enoblituzumab reported objective responses in prostate, melanoma, and bladder cancers [7]. A phase II study included patients with localized prostate cancer treated with enoblituzumab as neoadjuvant therapy and is ongoing (NCT06014255) [67]. Enoblituzumab has demonstrated a relatively high safety profile in early-phase trials, with side effects primarily consisting of infusion-related reactions, skin and gastrointestinal toxicities, and transient elevations of aminotransferases. These side effects appear to be minimal, likely due to the highly cancer-specific expression of B7H3 and its low prevalence in normal tissues. Most side effects were graded as 1 or 2 on the CTCAE scale, indicating mild to moderate reactions [68]. In contrast, anti-B7H3 CAR T-cell therapies exhibited a broader range of adverse effects, including Cytokine Release Syndrome (CRS), although there have been no reported cases of CRS-associated fatalities. Patients frequently experienced neurotoxic effects, primarily headaches and nausea. While on-target, off-target toxicity—often seen in CAR T therapies—can occur when modified lymphocytes damage healthy tissues expressing the targeted antigen; instances of this toxicity have not yet been reported in B7H3 CAR T therapy. On the contrary, anti-B7H3 CAR T-cell therapies demonstrated a higher diversity of adverse effects, particularly involving Cytokine Release Syndrome (CRS), without any reported cases of CRS-associated mortality. Neurotoxicity primarily manifested as headaches and nausea. On-target and off-target toxicity, a characteristic of CAR T-cell therapy that results from the damage to healthy tissue expressing the targeted antigen by modified lymphocytes, is a potential concern during B7H3 CAR T-cell therapy [69,70]. However, due to the very low expression of this immune checkpoint protein in normal tissue, reports of such toxicity have not yet emerged, as this therapy is still under active investigation. It is possible that as more clinical trials evaluating anti-B7H3 CAR T-cell therapies are completed, we may gain further insights into these effects. Targeting B7H3 may be a promising therapeutic option for patients with tumors that are resistant to PD-1/PD-L1 blockade, particularly in colorectal cancer (CRC) that expresses PD-L1 only in microsatellite instable tumors, which make up less than 15% of all cases [71]. In this context, B7H3 may serve as a prognostic and predictive marker for evaluating potential responses to immunotherapy in malignancies with low PD-1/PD-L1 levels, thereby broadening the population of patients who could benefit from effective treatment. Strategies that combine therapies specifically designed to inhibit B7H3, such as CAR T cells, with other modalities that enhance antitumor responses appear to be a promising approach for managing patients with more aggressive neoplasms.

The prognostic potential of B7H3 has been investigated in numerous studies; however, the results have often been inconsistent and contradictory. Therefore, we aimed to update and summarize the findings published thus far to draw reasonable conclusions. In total, 51 studies and 12,111 cases were included in the meta-analysis. The results demonstrated that B7H3 overexpression is a poor predictor of all analyzed clinical outcomes, including overall survival (OS), disease-free survival (DFS), progression-free survival (PFS), recurrence-free survival (RFS), and disease-specific survival (DSS).

Although Egger’s and Begg’s tests indicated a risk of publication bias, we did not perform adjustments due to the high heterogeneity among the included studies, which could make funnel plot assessment ineffective for detecting publication bias [72]. Sensitivity analyses showed that the results were stable, and excluding specific studies did not significantly affect the overall findings. Subgroup analyses confirmed both within-study and between-study heterogeneity. Alba et al. reported that meta-analyses pooling continuous outcomes, such as patient survival, show significantly higher I^2^ values compared to meta-analyses of binary outcomes. Additionally, the I^2^ in continuous outcome meta-analyses increases with the number of studies included. This suggests that it might be more appropriate to establish a different standard for interpreting I^2^ values [73]. The subgroup analysis for overall survival (OS), stratified by cancer type, sample size, and method for hazard ratio (HR) estimation, did not significantly reduce heterogeneity among the studies. However, an analysis based on cancer type revealed that B7H3 was associated with shorter OS in CC, ICC, OC, GC, NB, GEA, PTDC, CRC, HCC, PDAC, CC, but not in NSCLC, UCC, PCa, OC, ATC, RCC, SC, CP, and OS. The variations in these findings may stem from the distinct characteristics of different types of cancer, differences in study design, variations in expression estimation, a limited number of studies for certain cancers, and studies with small sample sizes, which resulted in pooled effect sizes with lower precision. In the subgroup analysis based on the method of HR estimation, a significant difference was noted between univariate and multivariate models, with the effect size being higher in studies that utilized multivariate HR estimates.

Analysis grouped by cut-off value for low and high B7H3 expression was difficult due to the very wide variety of methods used for B7H3 expression evaluation. Since the subgroup analysis based on cutoff values was inconclusive, we conducted an additional analysis comparing the percentage of positive cases in each study that could be linked to established cutoffs. However, this new comparison did not reduce the heterogeneity observed. As the application of anti-PD-1/PD-L1 therapy across various solid tumors has increased in recent years, detailed criteria for evaluating PD-L1 expression through immunohistochemistry have been established for specific cancers, making PD-L1 the most validated and used immune checkpoint. Even these criteria vary by tumor type and utilize different scoring algorithms, including the combined positive score (CPS), the tumor proportion score (TPS), and the tumor cells and immune cells score (TC and IC score) to assess PD-L1 positivity. The CPS measures the number of all cells that test positive for PD-L1 staining, which includes both tumor and immune cells, whereas the TPS evaluates PD-L1 staining exclusively in tumor cells. Additionally, the cutoff values for utilizing anti-PD-L1 agents differ by cancer type: for Pembrolizumab (anti PD-1 monoclonal antibody), TPS ≥ 1% is required for non-small cell lung cancer (NSCLC), CPS ≥ 1 for head and neck squamous cell carcinoma (HNSCC) and cervical cancer, and for patients with triple-negative breast cancer and esophageal or gastroesophageal junction tumors, a CPS greater than 10 is necessary. Consequently, even for such an extensively studied immune checkpoint, comparing the prevalence of upregulated expression across different tumors could yield inconclusive results. Currently, there is no standardized method for assessing B7H3 expression using IHC [74]. Most studies included in the meta-analysis have focused on B7H3 expression solely in tumor cells, employing H-scores calculated from the percentage of positive cells multiplied by staining intensity. However, the establishment of cutoff values to distinguish between tumors with high and low B7H3 expression remains inconsistent. This lack of standardization complicates the comparison of different studies and could be a significant source of heterogeneity.

We conducted further subgroup analyses stratified by sample size, the quality of studies assessed using the NOS, the method for estimating hazard ratios, publication year, and ethnicity. Our findings indicated that high B7H3 expression was associated with reduced OS only in Asian populations, while the association remained insignificant in Caucasians. Most studies were conducted in Asian countries, highlighting the need for further research to explore the prognostic potential of B7H3 in other populations due to possible genetic differences.

Additionally, we did not account for other factors that might influence the results, such as changes in cancer treatment, including the increasing number of approved immunotherapies in recent years. A comparison between studies published before and after 2020 did not yield significant differences in pooled HR or heterogeneity.

Since the source of high heterogeneity was not identified through subgroup and sensitivity analyses, we employed GOSH diagnostics to identify potential outlier studies. After excluding them, the heterogeneity in the meta-analyses pooling OS in solid tumors and gastrointestinal cancers was significantly reduced, while the pooled HR remained similar. The relationship between B7H3 and patients survival was investigated by previous meta-analysis. In 2016, Ye Z. et al. summarized results from 24 studies involving 4141 individuals and demonstrated that high expression of B7H3 is associated with shorter OS and RFS but not PFS. Furthermore, there was no significant difference in heterogeneity among the groups when stratified by cancer type, method of hazard ratio (HR) estimation, and sample size [75]. Su H conducted a meta-analysis exclusively for gynecological cancers, revealing a significant association with overall survival (OS), including 10 studies and 840 patients [76]. Similar studies were conducted separately for HNSCC and bladder cancer. The number of patients in these studies ranged from 1417 to 1622. B7H3 was identified as a risk factor for overall survival (OS) in HNSCC, but not in bladder cancer [77,78].

The study had several potential limitations. First, high heterogeneity was identified among the included studies, and the source of this heterogeneity was not fully determined through subgroup analysis or one-leave meta-analysis. It was reduced only after removing studies classified as potential outliers. This uncertainty affects the interpretation of results and complicates the assessment of publication bias. Second, the studies did not consistently establish a cut-off value for high B7H3 expression, which could significantly impact the reliability of B7H3 as a prognostic factor for cancer. Additionally, many studies failed to provide essential survival parameters needed for conducting a survival meta-analysis, such as HR or Kaplan–Meier curves along with a number-at-risk table. As a result, these studies could not be included in the analysis.

Finally, the inclusion criteria permitted only published articles and studies in English, which means that articles in other languages may have been excluded, contributing to potential selection bias.

The limitations of the study highlight the need for further high-quality research that employs standardized methods to evaluate B7H3 expression. It is also essential to establish consistent cut-off values to differentiate between high and low B7H3 expression. Such efforts are necessary to better understand the relationship between B7H3 expression and survival outcomes, as well as to more accurately identify the patient populations that could benefit from targeted B7H3 therapies.

## 5. Conclusions and Future Research Directions

Our results indicate that overexpression of B7H3 is significantly associated with poorer outcomes across all survival parameters, including OS, DFS, PFS, RFS, and DSS. This suggests that B7H3 has promising prognostic potential in solid tumors. However, the lack of standardized methods for assessing B7H3 expression, such as inconsistent cutoff values and varying immunohistochemical techniques, presents a major barrier to the generalizability and reproducibility of these findings. To provide a more comprehensive analysis, it is crucial to establish clear pathological guidelines for B7H3 testing via immunohistochemistry (IHC) across different tumor types. These guidelines should outline standardized immunostaining techniques, the evaluation and interpretation of scoring algorithms, and the thresholds for categorizing B7H3 expression as high or low, akin to the standards developed for assessing PD-L1 expression routinely used in clinical practice to qualify patients for therapies targeting PD-1/PD-L1. Despite extensive investigation into B7H3 in clinical trials, its evaluation remains significantly less advanced than that of PD-L1. Therefore, we can anticipate that standardized testing guidelines will emerge once anti-B7H3 therapies receive approval. Ongoing and future clinical trials focusing on B7H3 inhibition should establish precise methodologies for assessing B7H3 using IHC and report the cut-off values employed for stratifying B7H3 expression. Collaboration between researchers and pathologists is crucial not only for B7H3 but also for other immune checkpoints, including B7H4, HHLA2, TIGIT, VISTA, and ICOS/ICOS-L, which are under active evaluation. The development of common guidelines for assessing immune checkpoint expression via IHC is key to achieving validated findings that are comparable across studies and tumor types.

Given the high heterogeneity and limitations of the included studies, which complicate the assessment of publication bias, the findings should be interpreted with caution. Future research should prioritize the development of standardized criteria for B7H3 evaluation and focus on prospective multicenter studies to validate its prognostic significance, especially in non-Asian populations, as the majority of included studies were conducted in Asian countries. Overall, the integration of B7H3 into clinical practice as a biomarker or therapeutic target warrants further exploration, particularly in the context of combination therapies and immunotherapy-resistant tumors.

## Figures and Tables

**Table 1:** Characteristics of included studies. Abbreviations: Immunohistochemistry (IHC), overall survival (OS), progression-free survival (PFS), disease-free survival (DFS), disease-specific survival (DSS), recurrence-free survival (RFS), PCa—prostate cancer, OC—ovarian cancer, CRC—colorectal cancer, BC—breast cancer, NSCLC—non-small-cell lung carcinoma, GC—gastric cancer, LUAD—lung adenocarcinoma, EC—endometrial cancer, ATC—anaplastic thyroid cancer, PDTC—poorly differentiated thyroid carcinoma, PDAC—pancreatic ductal adenocarcinoma, UTUC—upper tract urothelial carcinoma, ACC—adrenocortical carcinoma, ICC—intrahepatic cholangiocarcinoma, HCC—hepatocellular carcinoma, ccRCC—clear cell renal cell carcinoma, UCC—urothelial cell carcinoma, HNSCC—head and neck squamous cell carcinoma, NB—neuroblastoma, PTC—papillary thyroid carcinoma, BLCA—bladder urothelial carcinoma, GBC—gallbladder cancer, CC—cervical cancer, GEA—gastroesophageal adenocarcinoma, SC—spinal chondroma, OS—osteosarcoma, CP—craniopharyngioma, AAC—ampullary adenocarcinoma, NOS—Newcastle–Ottawa Scale.

Author	Year	Patient Source	Sample Size	Method	Cancer Type	B7H3 + Expression	Outcome	HR	Multivariate/Univariate	Cohort/Subgroup	Cell Types	Cutoff	NOS
Guo C [14]	2023	United Kingdom	98	IHC	PCa	91	OS	Reported	U	CRPC	tumor cells	H score ≥ 5.	6
Guo C [14]	2023	United Kingdom	72	IHC	PCa	70	OS	Reported	U	HSPC	tumor cells	H score ≥ 5.	6
Miyamoto T [15]	2021	Japan	62	IHC	OC	31	OS/PFS	Reported	U/M		tumor cells	IHC score ≥ 2. B7H3 expression was graded from 0 (no staining) to 3 (strong staining intensity).	8
Wu J [16]	2018	China	225	IHC	CRC	197	OS	Reported	M		tumor cells	>5%	7
Joshi V [17]	2024	Australia	347	IHC	BC	174	DSS	Reported	M		tumor cells	>1%	8
Yonesaka K [18]	2018	Japan	82	IHC	NSCLC	60	PFS	Reported	M		tumor cells	>10%	7
Chen S [19]	2023	China	268	IHC	GC	180	OS	Reported	U		tumor and immune cells	Histoscore ≥ 2 (>10% positive cells).	6
Omori S [12]	2023	Japan	71	IHC	LUAD	31	OS	Pooled from Kaplan–Meier curve	U	Stage IV of disease	tumor cells	IHC score ≥ 2.	6
Amori G [20]	2021	Japan	135	IHC	PCa	41	OS/DFS/DSS	Reported	U/M		tumor cells	≥50% cells with moderate or strong staining.	8
Ingebrigtsen VA [13]	2014	Norway	562	IHC	CRC	162	OS/RFS	Pooled from Kaplan–Meier curve	U		tumor cells	Presence of B7H3 staining.	7
Inamura K [21]	2017	Japan	270	IHC	LUAD	86	OS/DSS	Reported	U/M		tumor cells	>50% postive cells with staining intensity 1 or >10% positive cells with staining intensity ≥ 2. Intensity of staining was classified as 0 (lack), 1 (weak or moderate) and 2 (strong).	8
Zong L [22]	2023	China	664	IHC	EC	598	RFS/DSS	Reported	U	tumor cells	tumor cells	Presence of any B7H3 staining	7
Zong L [22]	2023	China	664	IHC	EC	277	RFS/DSS	Reported	U	immune cells	immune cells	>1%	7
Luo Y [23]	2022	China	22	IHC	TC	19	OS	Reported	U	ATC	tumor cells and TILs	CPS ≥ 30. Tumor-infiltrating immune cells (TIICs) and cancer cells were stratified according to the combined positive score (CPS), classified as the percentage of positive cancer cells (total or partial membrane staining) and tumor TIICs (membrane or cytoplasm staining) divided by the total amount of tumor cells. The median CPS of cores of each specimen was evaluated as the final CPS. According to ICP, B7H3 expression was graded as negative (CPS < 1), weak (1 ≤ CPS < 10), moderate (10 ≤ CPS < 30), or strong (CPS ≥ 30).	6
Luo Y [23]	2022	China	44	IHC	TC	24	OS	Reported	U/M	PDTC	tumor cells and TILs	CPS ≥ 30. Detailed description of IHC evaluation above.	7
Xu YH [24]	2010	China	102	IHC	NSCLC	71	OS	Reported	M		tumor and noncancerous cells	>10%	6
Yang Y [25]	2022	China	56	IHC	LUAD	25	OS/PFS	Reported	U		tumor cells	Histochemical score > 6 for the B7H3 high group. Histochemical score ranged from 0 to 12 and was calculated as staining intensity (0—absent, 1—weak, 2—moderate, 3—strong) multiplied by the percentage of positive cells stratified into 0 (<5%), 1 (5–25%), 2 (26–50%), 3 (51–75%), and 4 (>75%).	6
Lee JH [26]	2023	South Korea	244	IHC	LUAD	73	PFS	Reported	U/M		tumor cells	≥50%	8
Chen X [27]	2023	China	240	IHC	PDAC	195	PFS/DSS	Reported	M		tumor cells	≥5%	8
Koyama Y [28]	2020	Japan	271	IHC	UTUC	235	PFS/DSS	Reported	U/M		tumor cells	≥50%	8
Liang J [29]	2020	China	48	IHC	ACC	44	OS/DFS	Reported	U/M		tumor cells	Presence of moderate or strong staining. The H-score of the cytoplasmic staining in cancer cells was graded as negative (0), weak (1), moderate (2) and strong (3).	8
Cheng R [30]	2018	China	45	IHC	ICC	26	OS/DSS	Reported	U/M		tumor cells	Final score ≥ 2 for B7H3 positive expression. Score ranged from 0 to 9 and was calculated as staining intensity (0—absent, 1—weak, 2—moderate, 3—strong) multiplied by the percentage of positive cells stratified into 1 (≤10%), 2 (10–50%), 3 (26–50%), and 4 (≥50%)	8
Zhou Z [31]	2023	China	61	IHC	HCC	33	RFS	Reported	U/M		tumor cells	The cutoff for the final score was determined by the ROC curve. The final score was calculated as staining density graded as 0 (≤5% positive cells), 1 (5–33%), 2 (33–66%), and 3 (>66%) assessed two times corresponding to five visual fields. Next, the two obtained scores were multiplied and averaged.	7
Bearrick EN [32]	2021	United States	436	IHC	ccRCC	234	DSS	Reported	U/M		tumor cells	>10%	8
Sun TW [33]	2012	China	240	IHC	HCC	168	DFS/RFS	Reported	M	study cohort	tumor cells	Staining intensity score ≥ 2. Staining was stratified into negative (0), weak (1), moderate (2), and strong (3). The percentage of positive cells was not evaluated.	8
Sun TW [33]	2012	China	205	IHC	HCC	205	OS/DFS/RFS	Reported	M	validation cohort	tumor cells	Staining intensity score ≥ 2. Staining was stratified into negative (0), weak (1), moderate (2), and strong (3). The percentage of positive cells was not evaluated.	8
Boorjian SA [34]	2008	United States	314	IHC	UCC	222	OS/PFS/DSS	Reported	U		tumor and adjacent noncancerous cells	>10%	7
Xylinas E [35]	2014	NA	302	IHC	UCC	177	OS/RFS/DSS	Reported	U		tumor and adjacent noncancerous cells	>10%	7
Katayama A [36]	2011	Japan	37	IHC	HNSCC	8	DSS	Reported	M		tumor cells	Staining intensity score ≥ 2. Scores were graded as 0—<5% positive cells, 1—weak, 2—moderate, and 3—strong. The percentage of positive cells was not analyzed.	8
Saeednejad Zanjani L [37]	2020	Iran	222	IHC	ccRCC	218	DSS	Reported	U/M		tumor cells	H score > 200 for high B7H3 expression. H score ranged from 0 to 300 and was calculated as staining intensity (0—negative, 1—weak, 2—moderate, 3—strong) multiplied by the percentage of positive cells classified as 1 (<25%), 2 (25–50%), 3 (51–75%), and 4 (≥50%).	8
Zeng L [38]	2023	China	212	IHC	NB	62	OS	Reported	U	Discovery cohort	NA	NA	8
Zeng L [38]	2023	China	272	IHC	NB	82	OS	Reported	U	validation cohort	NA	NA	8
Zhao B [39]	2022	China	343	IHC	PTC	211	RFS	Reported	U/M		tumor cells	Final score ≥ 2. It was evaluated as follows: 0—negative membranous staining or <1% cells with weak membranous staining, 1—≥1% cells with weak membranous staining or <1% cells with strong membranous staining, 3—≥1% cells with strong membranous staining.	8
Inamura K [40]	2019	Japan	252	IHC	ccRCC	99	OS/DSS	Reported	U/M		tumor cells	≥50% cells with moderate or strong staining intensity. Staining in cancer cell membranes was stratified into two groups: absent/weak and moderate/strong.	8
Asakawa A [41]	2024	Japan	103	IHC	LSCC	46	OS	Reported	M		tumor cells	>30%	7
Zhou Q [42]	2021	China	126	IHC	PCa	64	OS/DSS	Reported	U/M	training cohort	tumor cells	H score > 120 for high B7H3 expression determined by X-tile software 3.6.1. H score ranged from 0 to 300 and was calculated as staining intensity (0—negative, 1—weak, 2—moderate, 3—strong) multiplied by the percentage of positively stained cells.	8
Zhou Q [42]	2021	China	113	IHC	PCa	42	OS/DSS	Reported	U/M	validation cohort	tumor cells	H score > 120 for high B7H3 expression determined by X-tile software. H score ranged from 0 to 300 and was calculated as staining intensity (0—negative, 1—weak, 2—moderate, 3—strong) multiplied by the percentage of positively stained cells.	8
Nunes-Xavier CE [43]	2021	Norway, Spain. Survival analysis is available for the Norwegian cohort only.	206	IHC	PCa	78	RFS	Reported	U/M	Norwegian cohort	tumor cells	Presence of moderate to strong staining intensity.	8
Mahmoud AM [44]	2022	United States	81	IHC	BLCA	17	OS/RFS/DSS	Reported	U		tumor cells	Standardized H score > 120 for high B7H3 expression. H score ranged from 0 to 300 and was calculated as staining intensity (0—negative, 1—weak, 2—moderate, 3—strong) multiplied by the percentage of positively stained cells. The standardized H score was determined by using the average of H-scores obtained from two pathologists.	7
Ingebrigtsen VA [45]	2012	Norway	238	IHC	CRC	73	OS	Reported	U	colorectal cancer cohort	tumor cells	>10%	6
Ingebrigtsen VA [45]	2012	Norway	162	IHC	CRC	110	OS/DSS	Reported	U/M	colon cancer cohort	tumor cells	>10%	8
Lv C [46]	2022	China	95	IHC	GBC	67	OS/DSS	Reported	U/M	training cohort	tumor cells	H score > 60 for high B7H3 expression determined by X-tile software. H score was calculated as staining intensity (0—negative, 1—weak, 2—moderate, 3—strong) multiplied by the percentage of positively stained cells.	8
Lv C [46]	2022	China	103	IHC	GBC	70	OS/DSS	Reported	U/M	testing cohort	tumor cells	H score > 60 for high B7H3 expression determined by X-tile software. H score was calculated as staining intensity (0—negative, 1—weak, 2—moderate, 3—strong) multiplied by the percentage of positively stained cells.	8
Li Y [47]	2017	China	90	IHC	CC	33	OS	Reported	M	validation cohort	tumor cells	Final score ≥ 4 for positive B7H3 expression. The final score ranged from 0 to 7 and was calculated as the sum of positively stained cell percentages stratified into 0 (no positive cells), 1 (1–25% positive cells), 2 (26–50% positive cells), 3 (51–75% positive cells), and 4 (76–100% positive cells), and staining intensity (0—negative, 1—weak, 2—moderate, 3—strong).	7
Yim J [48]	2016	South Korea	484	IHC	NSCLC	190	OS	Reported	U/M		tumor cells	>25%	7
Zhang T [49]	2018	China	223	IHC	CRC	157	OS	Reported	U/M		tumor cells	Total score ≥ 4 for high B7H3 expression. Total score ranged from 0 to 12 and was calculated as staining intensity (0—negative, 1—weakly positive, 2—moderately positive, 3—strongly positive) multiplied by the percentage of positive cells divided into 0 (≤ 5%), 1 (6–25%), 2 (26–50%), 3 (51–75%), and 4 (> 76%).	7
Sun Y [50]	2024	China	57	IHC	GEA	28	OS/RFS	Reported	U		tumor cells	>1%	7
Xia C [51]	2022	China	92	IHC	SC	58	OS	Reported	U/M		stromal cells	Quantitative Immunofluorescence score (QIF) calculated as pixel intensities divided by the area of the corresponding mask. The cutoff value was determined using the Cutoff Finder Web Application.	7
Mao Y [52]	2015	China	128	IHC	NSCLC	89	OS	Reported	U/M		tumor cells	Total score ≥ 2 for positive B7H3 expression. Total score ranged from 0 to 9 and was calculated as staining intensity (0—negative, 1—weak, 2—moderate, 3—strong) multiplied by the percentage of positive cells graded as 0 (no positive cells), 1 (1–10%), 2 (11–50%), and 3 (>50%).	7
Maeda N [53]	2014	Japan	90	IHC	PCa	64	RFS	Reported	M		tumor cells	Final score ≥ 2 for high B7H3 expression. Final score ranged from 1 to 6 and was calculated as staining intensity (absent/weak staining—1, moderately intense staining—2, strong staining—3) multiplied by the percentage of positive scored as <33% of cancer cells—1, ≥33 to 66% of cancer cells—2, and >66% of cancer cells—3.	8
Zang X [54]	2007	United States	803	IHC	PCa	212	RFS/DSS	Reported	U		tumor cells	Strong staining intensity (complete membranous expression). IHC intensity was divided into none, weak, moderate, or strong according to the strength of membranous staining.	7
Xie L [55]	2023	China	35	IHC	OS	10	OS/PFS	Reported	U		tumor cells	>1%	7
Lu Z [56]	2022	China	805	IHC	CRC	410	OS/DFS	Reported	M		tumor cells	>1%	8
Wang Y [57]	2020	China	132	IHC	CP	45	OS/PFS	Reported	U/M		tumor cells	NA	7
Inamura K [58]	2018	Japan	150	IHC	PDAC	99	OS/DFS	Reported	U/M		tumor cells	>10%	8
Geerdes EE [59]	2021	Netherlands	83	IHC	AAC	55	RFS/DSS	Reported	U/M		tumor cells	NA	8
Iida K [60]	2019	Japan	87	IHC	RCC	51	DFS	Reported	U/M		tumor cells	>10%	8
Chen L [61]	2020	China	74	IHC	HCC	38	OS	Reported	U		tumor cells	IHC score ≥ 4. Score ranged from 0 to 9 and was determined by multiplication of staining intensity categorized into 0—negative, 1–weak, 2—moderate, and 3—strong and the percentage of positive cells graded as 0 (no positive cells), 1 (<40%), 2 (40–70%), and 3 (>70%).	6

## Data Availability

The original contributions presented in the study are included in the article, further inquiries can be directed to the corresponding author/s.
